# Integrated analysis of bulk and single‐cell RNA‐seq data reveals cell differentiation‐related subtypes and a scoring system in bladder cancer

**DOI:** 10.1111/jcmm.70111

**Published:** 2024-10-14

**Authors:** Sheng Li, Fucun Zheng, Zhipeng Wang, Situ Xiong, Jin Zeng, Songhui Xu, Bin Fu, Xiaoqiang Liu

**Affiliations:** ^1^ Department of Urology The First Affiliated Hospital of Nanchang University Nanchang China; ^2^ The First Affiliated Hospital of Nanchang University Nanchang China; ^3^ Key Laboratory of Urinary System Diseases of Jiangxi Province Nanchang China

**Keywords:** bladder cancer, cell differentiation, single‐cell data, subtypes, WGCNA

## Abstract

Bladder cancer (BLCA) exhibits notable molecular heterogeneity, influencing diverse clinical outcomes. However, the molecular subtypes associated with cell differentiation‐related genes (CDR) and their prognostic implications remain unexplored. Analysing two GEO single‐cell datasets, we identified genes linked to cell differentiation. Utilizing these genes, we explored distinct molecular subtypes. WGCNA analysis further identified CDR‐associated genes, and the CDR score system, constructed using Lasso and Cox regression, was developed. Clinical prognosis and variations in immune‐related factors among patient groups were assessed. Core genes were selected and confirmed through in vitro experiments. Two BLCA subtypes related to cell differentiation were identified: Subtype B demonstrated a favourable prognosis, while Subtype A exhibited significant immune cell infiltration. The CDR score system of nine genes revealed a positive correlation between higher scores and a poorer prognosis. The comprehensive analysis uncovered a positive association between CDR genes and M2 macrophages and unresponsiveness to immune therapy. Functional experiments validated that ANXA5 downregulation influences tumour cell migration without affecting proliferation. Our study reveals distinct cell differentiation‐related molecular subtypes and introduces the CDR scoring system in BLCA. ANXA5 emerges as a potential therapeutic target, offering promising avenues for personalized treatment strategies.

## 1 INTRODUCTION

In 2020, bladder and kidney cancers collectively accounted for over one million incident cases and approximately 400,000 new fatalities worldwide.^1^ Although many cases are detected at an early stage, with a 5‐year survival rate reaching about 95%, many patients experience relapse within this period, resulting in an aggressive disease course and a considerable reduction in life expectancy.^2^, ^3^ Bladder cancer (BLCA) is associated with the highest therapeutic costs among all cancer types in the United States and Europe, significantly impacting public health expenditures.^4^, ^5^ Consequently, there is a pressing need for efficacious targeted treatments to facilitate patient outcomes while mitigating healthcare expenses. In recent times, considerable research endeavours have been dedicated to delineating molecular subtypes and biomarkers to enhance our understanding of the disease and facilitate personalized therapeutic strategies.

Cell differentiation plays a pivotal role in the initiation and progression of cancer. Altered expression and dysregulation of genes involved in cell differentiation have been implicated in tumorigenesis, tumour growth and metastasis across diverse malignancies. Previous studies have identified cell differentiation‐related genes as prognostic indicators in non‐small cell lung cancer,^6^ employed for classification and recurrence prediction in pituitary neuroendocrine tumours,^7^ and implicated in immune infiltration dynamics in gastric cancer.^8^ However, a certain knowledge gap remains regarding the precise molecular subtypes and prognostic implications of cell differentiation‐related genes (CDRs) in the context of BLCA.

Single‐cell analysis has revolutionized our comprehension of tumour heterogeneity, yielding unprecedented insights into the cellular composition and the immunological microenvironment within tumours.^9^, ^10^ Leveraging single‐cell sequencing data empowers researchers to model cell differentiation trajectories and identify genes associated with distinct differentiation stages, thus presenting new avenues to explore hitherto undiscovered cell differentiation‐related genes. Weighted Gene Co‐Expression Network Analysis (WGCNA) represents a powerful method for scrutinizing gene expression patterns across multiple samples. By clustering genes based on shared expression patterns to form modules, WGCNA facilitates the investigation of interrelationships between these modules and specific phenotypic features, such as tumour grading.^11^

**FIGURE 1:**
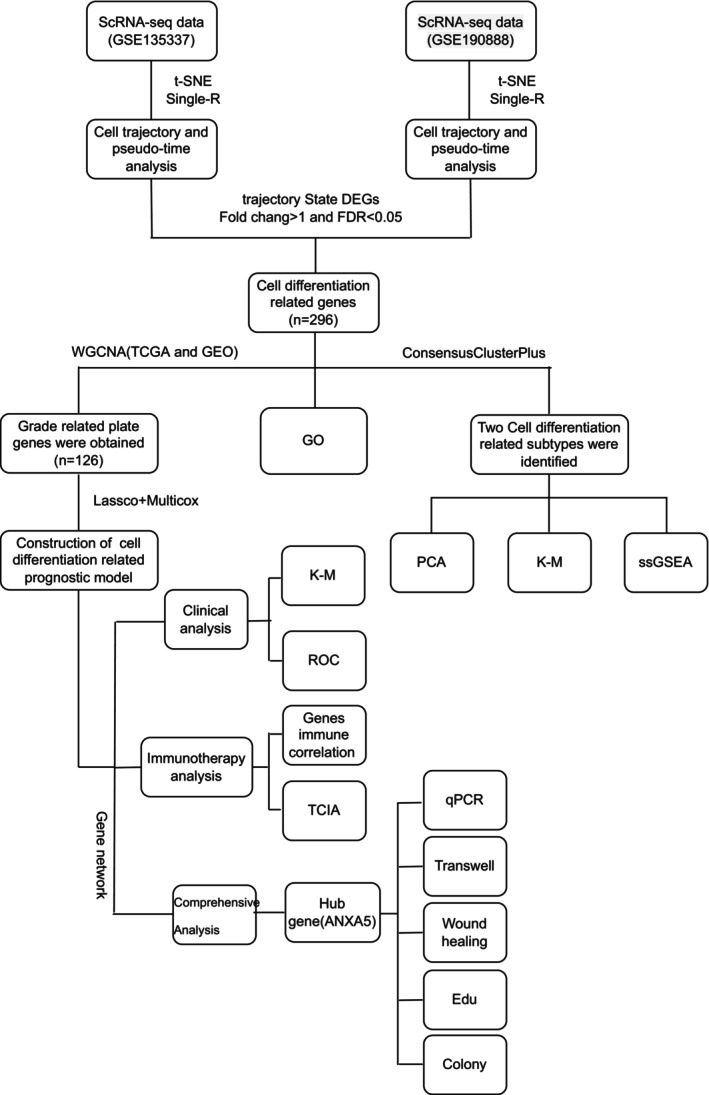
The entire process of this study.

In this study, cell differentiation‐related genes in BLCA were identified through single‐cell data analysis. Subsequently, patients were categorized into two distinct subtypes, revealing disparities in prognostic outcomes and immune infiltration patterns between these groups. Comprehensive WGCNA analyses were then performed on the identified cell differentiation genes, utilizing publicly available TCGA and GEO bladder cancer datasets, further uncovering genes closely linked to cell differentiation. Notably, a novel cell differentiation‐related (CDR) scoring system was developed to quantitatively assess the impact of CDRs on clinical outcomes and the immunological microenvironment. The pivotal gene, ANXA5, was identified as a promising therapeutic target for BLCA, with its potential validated through in vitro experiments. The entire research process is illustrated in Figure 1.

## 2 METHODS

### 2.1 Bladder cancer data acquisition

Two BLCA single‐cell datasets, GSE135337 and GSE190888 and one bladder cancer RNA sequencing dataset, GSE13507, were obtained from the Gene Expression Omnibus (GEO). Additionally, BLCA datasets were downloaded from The Cancer Genome Atlas (TCGA), and BLCA immunotherapy data were retrieved from The Cancer Immunome Database (TCIA, https://www.tcia.at/home) website. With the assistance of a Perl script, the TPM (Transcripts Per Million) data of BLCA from TCGA was extracted. Subsequently, the ‘SVA’ package in R software (version 4.3.1) was employed to eliminate batch effects, resulting in the generation of the merged dataset combining TCGA and GSE13057 data (Table S1).

### 2.2 Identification of bladder cancer cell differentiation‐related genes and gene ontology (GO) enrichment analysis

We applied the ‘Seurat’ package to transform the scRNA‐seq data into Seurat objects. To ensure the quality of the raw counts, we computed the percentage of mitochondrial or ribosomal genes and filtered out low‐quality cells. Subsequently, we employed the ‘FindVariableFeatures’ function to select the top 2000 highly variable genes after quality control. For dimensionality reduction and visualization, we implemented t‐distributed stochastic neighbour embedding (t‐SNE) techniques within the framework of Principal Component Analysis (PCA). To identify marker genes for each cluster, we used the FindClusters function from the Seurat package and determined the optimal resolution using the Clustree tool. Clustree helps us select an appropriate resolution for subsequent analysis by visualizing the interactions between different clusters at various resolutions. In addition, we used the FindAllMarkers function from the Seurat package to identify the top 5 most differentially expressed genes in each cluster and visualized them using a heatmap. Cell types were manually annotated to define distinct cellular populations. We conducted cell trajectory and pseudo‐time analyses using the ‘monocle’ package, focusing on the epithelial cells.

Differential analysis was carried out on diverse differentiation states, where we defined cell differentiation‐related marker genes based on criteria with |log Fold Change| > 0.585 and false discovery rates (FDR) < 0.05. The acquired cell differentiation‐related genes underwent GO enrichment analysis to further explore their biological significance.

### 2.3 Bladder cancer molecular subtype identification and prognosis

We used the merged dataset to identify molecular subtypes based on cell differentiation‐related genes. PCA was employed to validate the robustness of our clustering. Kaplan–Meier survival curves were utilized to compare the prognostic differences between the identified subtypes, and single‐sample Gene Set Enrichment Analysis (ssGSEA) was conducted to evaluate their distinct immune infiltration patterns.

### 2.4 Establishment of cell differentiation‐related (CDR) scoring system

Utilizing WGCNA, we generated module‐trait heatmaps to further identify module genes in BLCA tumour grading by comparing their correlation coefficients and *p*‐values. We opted to conduct subsequent analyses using the intersection of genes from the TCGA and GEO datasets that exhibited module correlation coefficients and a *p*‐value below 0.05. Subsequently, we constructed a scoring model using the Lasso and Cox regression: Score = $\sum_{i=1}^{n}\text{coef}*\text{Gene}\;\mathrm{Exp}$. The merged dataset was then split into training and validation sets at a 1:1 ratio. Based on the median score, BLCA patients were categorized into high and low‐scoring groups. Kaplan–Meier survival curves were employed to compare the survival outcomes between the two groups. Furthermore, we investigated the correlation between the scoring system and clinical pathological features while verifying the accuracy of the scoring model through ROC curves. Finally, we explored whether there was a correlation between CDR subtypes and CDR scoring systems, and visualized it through the ‘ggplot’ package.

### 2.5 Analysis of the correlation between the scoring system and the immune microenvironment

We used the VlnPlot function from the Seurat package to create violin plots for the datasets, which were used to display the expression levels of model genes. The distribution of these genes was externally validated using the GSE145281 BLCA single‐cell dataset from the TISCH2 online repository (http://tisch.comp‐genomics.org/home/). To access the dataset, visit the TISCH2 website, click on ‘BLCA’ to select BLCA, locate the GSE145281 dataset and navigate to the ‘Gene’ section. Enter the model genes to analyse their expression across various cell types. Moreover, the CIBERSORT algorithm was employed to quantify the correlation between model genes and immune cell populations. Lastly, we analysed the TCIA bladder cancer immunotherapy data to investigate variations in immunotherapy responsiveness among distinct groups.

### 2.6 Kaplan–Meier survival analysis and identification of hub genes

The impact of genes in the scoring system on the prognosis of BLCA patients was analysed using Kaplan–Meier survival curves (*p* < 0.05). Based on the coefficients of the scoring system genes, we selected the most optimal core genes.

### 2.7 Cell culture and transfection

Bladder cancer cell lines T24 (CL‐0227), 5637 (CL‐0002), J82 (CL‐0125), UMUC3 (CL‐0463) and SV‐HUC‐1 (CL‐0222) were obtained from Procell Life Science & Technology (Wuhan, China). 5637 and SV‐HUC‐1 cells were cultured in RPMI‐1640 supplemented with 10% FBS, while J82 and UMUC3 cells were cultured in MEM with 10% FBS. T24 cells were cultured in DMEM with 10% FBS. All cells were maintained in a humidified incubator at 37°C with 5% CO_2_. For targeted ANXA5 knockdown, we transiently transfected cells with specific ANXA5 siRNA using Lipofectamine 2000 (Invitrogen, USA). In our study, we designed two smalls interfering RNAs (siRNAs) targeting ANXA5, referred to as siRNA S1 and siRNA S2. The specific sequences for these siRNAs, as well as the sequence of the ANXA5 gene, are provided in Table S2.

### 2.8 RNA extraction and real‐time quantitative PCR (qRT‐PCR)

Ten pairs of BLCA tissues were collected from the First Affiliated Hospital of Nanchang University, with the study receiving ethical approval. Total RNA was extracted from the tissues using Invitrogen TRIzol reagent, and cDNA was synthesized using the Takara PrimeScript RT kit. RT‐qPCR was conducted using SYBR Green (Roche, Switzerland). The 2^‐ΔΔCt method was utilized to determine the relative expression levels of the genes, with ACTIN serving as the reference gene.

### 2.9 Cell proliferation assay

After transfecting T24 and BIU cells for 36 h, 1000 cells were seeded into each well of a 6‐well plate and cultured for 14 days. Subsequently, they were fixed with 4% paraformaldehyde for 20 min and stained with 1% crystal violet for 30 min. In addition, transfected cells were plated at a density of 5000 cells per well in a 96‐well plate. After 24 h, staining was performed following the instructions provided in the Edu assay kit (C10310‐1, RiboBio).

### 2.10 Wound‐healing and transwell assay

In the migration assay, transfected 40,000 cells were evenly seeded into the upper chamber. The upper room was filled with 200 μL of serum‐free medium, while the lower room contained 800 μL of medium with 20% FBS. After 36 h of incubation, the cells were fixed with methanol and stained with 0.1% crystal violet. Subsequently, cells on the lower surface of the chamber were captured under a microscope and counted to measure the extent of cell migration. Similarly, cells were seeded into 6‐well plates at a density of 60% and transfected with interfering fragments to reach 90% confluence. A 1 mL pipette tip was used to create a scratch on the cell monolayer, and any debris was washed away with PBS. Then, 1 mL of serum‐free medium was added to each well. Images were taken at 0 and 36 h after scratching, and the scratch area was quantified using ImageJ software.

### 2.11 Statistical analysis

All bioinformatics analyses were performed using R software, and experimental data were visualized using GraphPad Prism 7.0. Student's *t*‐test was used to compare the means between two groups, while one‐way/two‐way ANOVA was used to compare the means among multiple groups. A *p*‐value <0.05 was considered statistically significant.

## 3 RESULTS

### 3.1 Cell annotation and trajectory analysis

**FIGURE 2:**
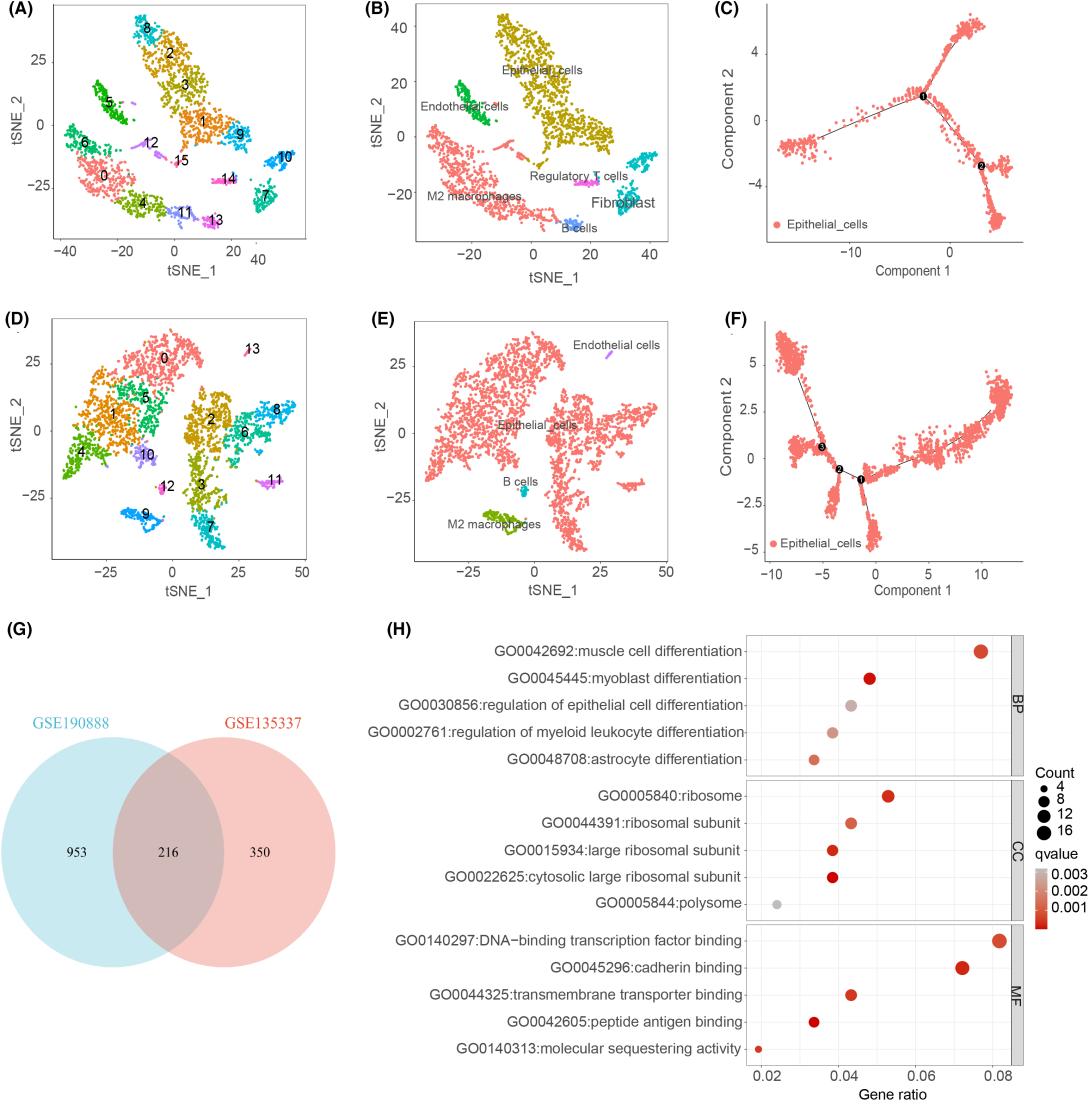
Analysing single‐cell RNA sequencing datasets derived from GSE190888 and GSE135337. Clusters annotation and cell types identification via tSNE in GSE190888 (A, B) and GSE135337 (D, E). Cell trajectory analysis for the identified epithelial cells in GSE190888 and GSE135337 (C, F). (G)Venn diagrams illustrate the overlap of CDR‐related genes identified in the two datasets, GSE190888 and GSE135337. (H). Gene Ontology (GO) analysis of CDR‐related genes was conducted to investigate the biological processes, molecular functions and cellular components associated with these genes.

Five single‐cell BLCA datasets were downloaded from GSE190888 and two from GSE135337. Following meticulous quality control, 6150 and 6167 cellular entities were discerned. The uppermost 2000 genes displaying remarkable variability (Figure S1A,B) were harnessed for ensuing principal component analysis (PCA) and t‐distributed stochastic neighbour embedding (t‐SNE) analyses. Based on the results from Clustree (Figure S1C,D), we set the resolution parameter of the FindClusters function to 1.0 for both datasets. While GSE190888 unveiled 16 distinct clusters, GSE135337 delineated 14 clusters (Figure 2A,D). Figure S2A,B show the top 5 most differentially expressed genes in each cluster for GSE190888 and GSE135337, respectively. Subsequently, manual clustering annotations were applied, utilizing marker genes extracted from the CellMarker database (http://xteam.xbio.top/CellMarker/) and visualization through t‐SNE plots.

In the GSE190888 dataset, we pinpointed six distinct cellular phenotypes: Epithelial cells, M2 macrophages, Endothelial cells, Fibroblasts, Regulatory T cells and B cells (Figure 2B). Analogously, in the GSE135337 dataset, four discernible cellular types emerged, namely Epithelial cells, M2 macrophages, Endothelial cells and B cells (Figure 2E). To glean preliminary insights into the genetic determinants underlying cell differentiation, we simulated discrete cellular trajectories within the two BLCA datasets, thereby diligently observing the phenomenon of cellular differentiation. In the GSE190888 dataset, the epithelial cell differentiation trajectory is depicted in Figure 2C, showing a trifurcating developmental path where cells diverge from a common starting point (marked as 0) into two primary branches (marked as 1 and 2). This suggests that the cells may differentiate into two distinct types. In contrast, Figure 2F of the GSE135337 dataset presents a more intricate trajectory with four key nodes (marked as 0, 1, 2 and 3), indicating that cells undergo multiple differentiation steps throughout their development. Following this, we conducted differential analysis on cells from these various branches to identify genes associated with cell differentiation.

### 3.2 Differentially expressed genes (DEGs) and gene ontology (GO) enrichment analysis

In the GSE190888 dataset, we discerned a total of 1169 genes exhibiting differential expression, while in the alternative dataset, 566 distinctively expressed genes were identified. To strengthen the reliability of our outcomes, we overlapped the two sets, resulting in a combined list of 216 genes potentially related to cellular differentiation (Figure 2G, Table S3). The outcomes of GO enrichment analysis showcased a pronounced enrichment of these differentially expressed genes in pathways attributed to the regulation of epithelial cell differentiation, the muscle cell differentiation and the myoblast differentiation (Figure 2H).

### 3.3 Identification of cell differentiation‐related (CDR) subtypes and comprehensive analysis

**FIGURE 3:**
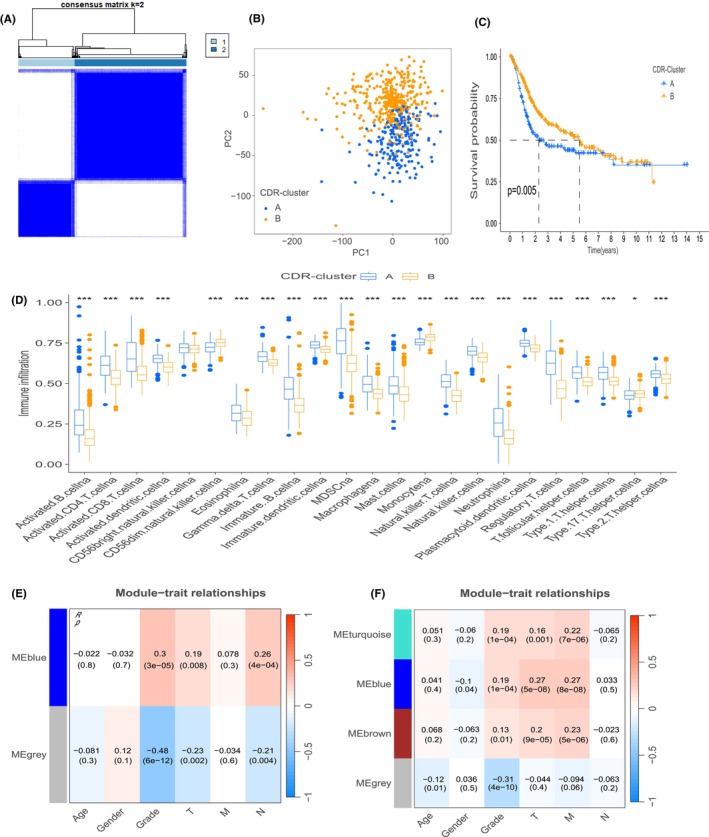
Cell Differentiation‐related (CDR) Molecular Subtype Identification and WGCNA analysis in bladder cancer. (A) Consensus cluster analysis indicated that the optimal number of clusters was *k* = 2, dividing patients into two distinct subgroups. (B) Principal Component Analysis (PCA) analysis of two distinct subtypes. (C) Kaplan–Meier survival analysis between subtype A and B. (D) Analysis of immune cell infiltration in the two CDR subtypes was performed using ssGSEA (single‐sample Gene Set Enrichment Analysis). Statistical significance was indicated as follows: *p < 0.05; **p < 0.01; ***p < 0.001. (E, F) The heatmap demonstrates the relationship between various gene modules and clinical characteristics in GSE13507 and TCGA.

After data manipulation, we aggregated RNA sequencing data from a cohort of 594 BLCA patients, drawing from both TCGA and GSE13507 datasets. Employing co‐clustering analysis, we discerned that an optimal partition into two clusters, mainly when k equals 2, aligns with the data. Consequently, we stratified BLCA patients into two distinctive CDR subtypes: A and B (Figure 3A). Notably, subtype A encompassed 200 instances, while subtype B comprised 394 cases (Table S4). Principal Component Analysis (PCA) underscored the robustness of our clustering methodology, graphically illustrating that patients can be segregated into two distinct cohorts based on our taxonomy (Figure 3B). Intriguingly, Kaplan–Meier survival analysis revealed a difference in prognosis, with subtype A having a worse outlook compared to subtype B (Figure 3C). Furthermore, we examined the relationship between subtypes and immune cell constituents. Interestingly, we found that the relative abundance of immune cells in subtype B is generally lower than in subtype A, with a few exceptions, such as CD56 dim NK cells and monocyte cells (Figure 3D).

### 3.4 Establishment and validation of CDR scoring system based on TCGA and GEO datasets

Cellular differentiation profoundly influences tumour grade, prompting our endeavour to extract genes associated with grade through the systematic analysis of Weighted Gene Co‐expression Network Analysis (WGCNA). This approach was further refined to identify genes intricately linked with cellular differentiation. Based on the principles of network independence and average connectivity within datasets, we meticulously selected a power value of 15 as the optimal threshold for soft thresholding within the GSE13507 dataset. Similarly, for the TCGA dataset, a power value of 3 was identified as the optimal threshold, as showcased in Figure S3A,B.

A discernible separation into distinctive blue and grey modules emerged in the former dataset. Our focus rested on the blue module, housing 126 genes exhibiting the highest, statistically positive correlation coefficients with Grade (Figure 3E, Table S5). In parallel, the latter dataset exhibited division into four distinct modules. Our scrutiny was concentrated on the turquoise and blue modules, collectively harbouring 220 genes (Figure 3F, Table S5), identifying a cumulative pool of 126 overlapping genes (Table S5). We then intersected these genes with those identified through single‐cell analysis, resulting in a total of 28 key genes associated with cell differentiation (Table S5).

**FIGURE 4:**
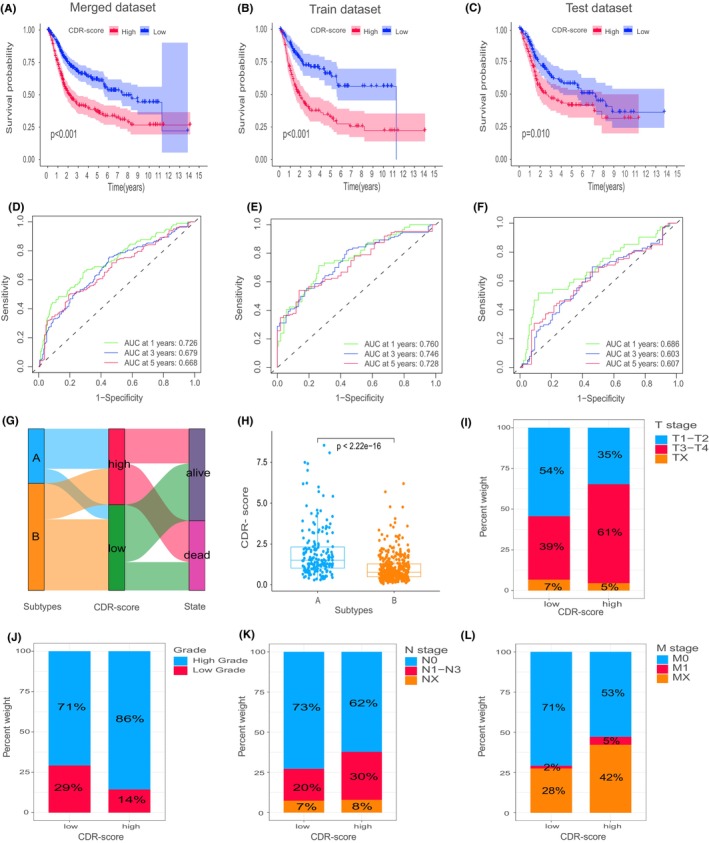
Clinical analysis of CDR scoring system. RNA sequencing data from 594 bladder cancer patients (TCGA and GSE13507) were combined, split into training and validation sets (1:1 ratio), and patients were categorized into high and low‐scoring groups based on the median score. K‐M survival analysis of CDR score in entire cohort (A), train cohort (B) and test cohort (C). ROC curves depicting the prediction of overall survival at 1, 3 and 5 years in the complete cohort (D), training subset (E) and test subset (F). Correlation between CDR subtypes and CDR scoring systems (G and H). Relationship between the scoring system and clinical pathological features (I‐L).

Employing Lasso + multivariable Cox regression analysis, we derived a scoring system characterized by the involvement of nine genes with a clear connection to cellular differentiation (Figure S3C,D): CDR score = (0.24424 * RGS2) + (−0.37577 * FGL2) + (−0.38439 * LST1) + (0.34542 * ANXA5) + (0.16455 * COL5A2) + (0.37821 * C1QB) + (0.24391 * IFITM2) + (−0.26355 * IGFBP7) + (−0.24680 * RGS1). Subsequent evaluation encompassing amalgamated, training and validation datasets demonstrated a correlation between elevated scores and a more unfavouraeble prognosis (Figure 4A–C). The Area Under the Curve (AUC) for the combined dataset at 1, 3 and 5 years amounted to 0.726, 0.679 and 0.668, respectively (Figure 4D). The AUCs for the training dataset were 0.760, 0.746 and 0.728, while the validation dataset exhibited corresponding values of 0.686, 0.603 and 0.607 (Figure 4E,F).

These findings indicate the prognostic potential embedded within our scoring system concerning BLCA. Patients in the higher‐scoring subgroup had higher T stages (T3‐T4, 61% vs. 39%), higher tumour grades (86% vs. 71%), higher N stages (N1‐N3, 30% vs. 20%) and fewer M0 stages (M0, 53% vs. 71%), attributing to the observed suboptimal prognosis within this particular subgroup (Figure 4I–L). There were no discernible differences observed among different groups based on gender and age (Figure S3E). The coherence of these findings validates the precision of our molecular classification and scoring approach. Figure 4G,H illustrate a correlation between CDR subtypes and the CDR scoring system, with subtype A corresponding to higher CDR scores.

### 3.5 CDR score exhibits a correlation with M2 macrophages and immunotherapeutic interventions

**FIGURE 5:**
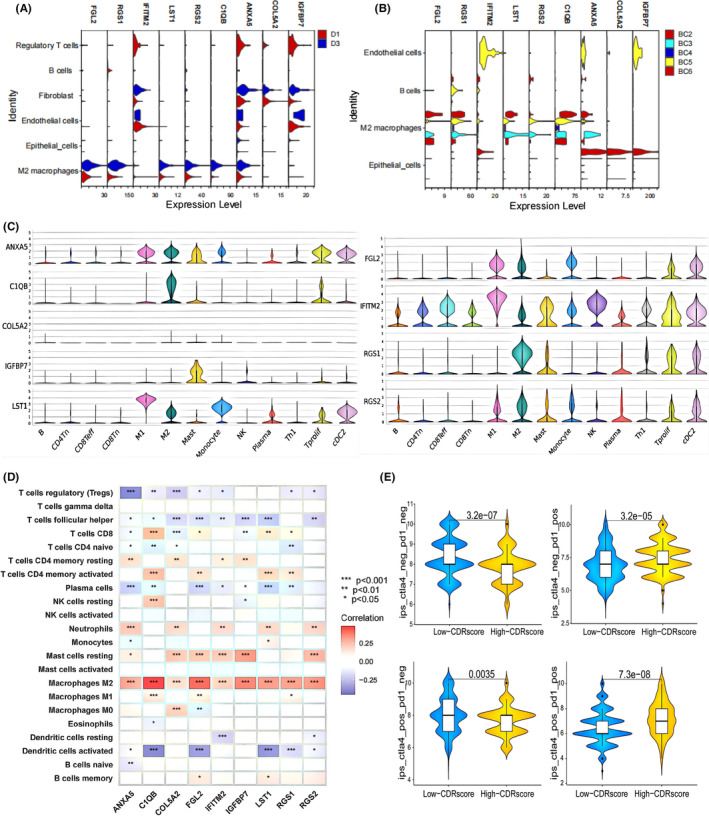
Correlation Between the Scoring System and the Immune Microenvironment. Three violin plots were used to visualize the expression of 9 genes related to the nine scoring system genes across the datasets GSE190888, GSE135337 and the external validation dataset GSE145281 (A, B, C). Correlation analysis between the scoring system genes and immune cells is shown in (D). Statistical significance was indicated as follows: *p < 0.05; **p < 0.01; ***p < 0.001. Analysis of immunotherapy sensitivity in both high and low‐scoring groups within the TCIA dataset is presented in (E). isp_ctla4_pos_pd1_neg refers to patients with positive CTLA4 expression and negative PD1 expression. isp_ctla4_neg_pd1_pos denotes patients with negative CTLA4 expression and positive PD1 expression. isp_ctla4_neg_pd1_neg represents patients with both CTLA4 and PD1 expression negative. isp_ctla4_pos_pd1_pos indicates patients with both CTLA4 and PD1 expression positive. Higher scores on the vertical axis represent greater sensitivity to immunotherapy.

As shown in Figure 5A–C, the majority of model genes are enriched in M2 macrophages across the three datasets. The outcomes derived from CIBERSORT analysis revealed that a substantial proportion of genes within the CDR scoring system exhibited positive correlations with M2 macrophages, neutrophils and Mast cells resting, while concurrently displaying inverse correlations with follicular helper T cells and regulatory T cells (Figure 5D). Results from the TCIA immunotherapy subgroup analysis reveal an interesting observation: in patients with positive PD1 expression, regardless of CTLA4 expression, the group with high CDR scores is more responsive to immunotherapy; conversely, in patients with negative PD1 expression, regardless of CTLA4 expression, the group with low CDR scores is more responsive to immunotherapy (Figure 5E).

### 3.6 Utilizing ANXA5 as the core gene of the scoring system and conducting in vitro validation

**FIGURE 6:**
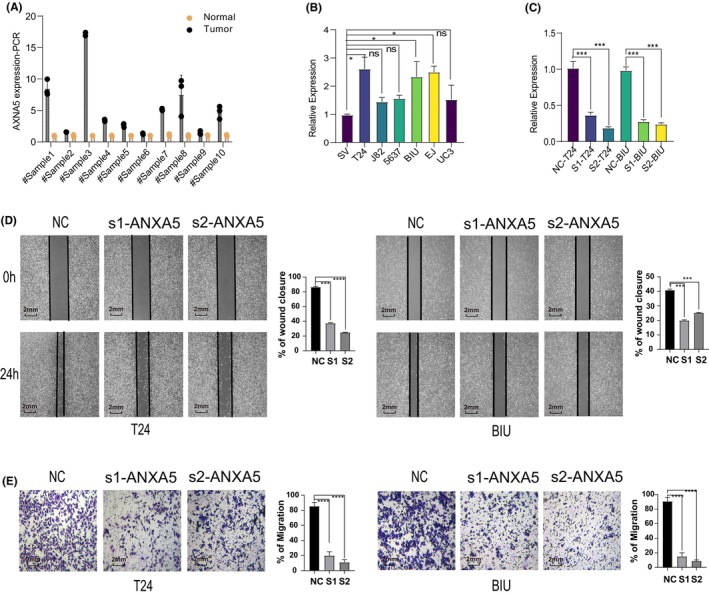
Functional Verification of Knockdown ANXA5. ANXA5 expression in bladder cancer tissues and cells was detected using qPCR, and the interference efficiency of small interfering RNA (siRNA) was also validated (A, B and C). Evaluation of cell migration ability using wound healing and transwell assays was conducted to assess the effect of ANXA5 knockdown on bladder cancer cell migration (D and E). Statistical significance was indicated as follows: *p < 0.05; **p < 0.01; ***p < 0.001; ****p < 0.0001.

K‐M survival analysis shows that patients with high expression of ANXA5, COL5A2, RGS2 and C1QB (*p* < 0.05) have worse prognosis, particularly ANXA5 and COL5A2, both with *p*‐values less than 0.001. Considering the coefficients from the scoring system described earlier, ANXA5 has a higher coefficient than COL5A2 (0.34542 vs. 0.16455). Therefore, ANXA5 may be an important risk factor influencing prognosis. We have chosen to conduct further experimental validation with ANXA5. Our analysis of BLCA patient tissues and cell lines using PCR techniques reveals overexpression of ANXA5 within both tumour tissues and cells (Figure 6A,B). Considering the increased ANXA5 expression in T24 and BIU cells, we selected these cell lines as the basis for our upcoming experimental pursuits. All normalized PCR data can be found in Table S6.

Initially, we validated the successful transfection of S1 and S2 within T24 and BIU cells via PCR assessments (Figure 6C). This validation procedure laid the groundwork for the execution of function‐oriented experiments harnessing the potency of S1 and S2. The outcomes stemming from the Transwell and wound healing assays indicate a reduction in the migratory prowess of BLCA cells, a direct result of suppressed ANXA5 expression (Figure 6D,E). Interestingly, knocking down ANXA5 did not result in a significant impact on the proliferation of T24 and BIU cells, as observed in both colony formation and Edu experiments (Figure S3G,H). In conclusion, these data demonstrate that ANXA5 influences the migration of BLCA cells.

## 4 DISCUSSION

Bladder carcinoma, a prevailing malignancy, demonstrates a propensity for swift progression and profoundly influences the patient's quality of life.^12^ Despite the potential for mitigating mortality risks via platinum‐based combinatorial therapeutic approaches, the aggregate responsiveness rate in patients remains suboptimal, falling short of the 50% threshold.^13^ Research indicates that complex pathological subtypes and the tumour microenvironment could lead to drug resistance and diverse prognoses.^14^, ^15^ Therefore, an imperative arises to delve into emergent molecular subtypes within bladder carcinoma and identify salient prognostic molecular biomarkers. In this research, we employed single‐cell and WGCNA analyses to pinpoint genes linked to BLCA cell differentiation. GO enrichment analysis revealed that these genes are predominantly enriched in pathways associated with cell differentiation, indicating the accuracy of our acquisition of cell differentiation‐related genes. We differentiated two patient subtypes exhibiting distinct prognostic and immune profiles through these genes. Employing Lasso + Cox regression, we developed the Cell Differentiation‐Related (CDR) scoring system, categorizing patients into high and low scores. Additionally, we delved into the clinical and immune correlations across these groups. Lastly, through comprehensive analysis, we identified ANXA5 as the pivotal gene within the scoring system and validated its potential as a promising therapeutic target for BLCA through laboratory validation.

Consensus clustering is an approach reliant on gene expression matrices for identifying distinct molecular subtypes.^16^ Two distinct BLCA subtypes linked to cell differentiation have been identified. Survival curves suggest that the prognosis for subtype A is poorer than that of subtype B. Analysis of immune cell infiltration reveals that, except for lower expression of CD56 dim NK cells and monocytes in subtype A, other immune cells show higher expression in this subtype.

We established a scoring system through Lasso + Cox regression analysis, comprising nine cell differentiation‐related genes: RGS2, RGS1, FGL2, LST1, COL5A2, IFITM2, C1QB, IGFBP7 and ANXA5. RGS2 (Regulator of G Protein Signalling 2) is pivotal in driving cancer recurrence and drug resistance through mechanisms involving dormant cells.^17^ Prior research has highlighted the role of RGS2 in the microRNA‐493‐3p‐mediated axis of BLCA progression.^18^ RGS1 is crucial for cancer immune evasion and is upregulated in multiple cancers. Song et al. found that high RGS1 reduces T cell infiltration and survival, indicating its potential as an immunotherapy target.^19^ Jiang et al. linked elevated RGS1 in gastric cancer to reduced cell proliferation and apoptosis, and noted its role in TAM activation and macrophage polarization, suggesting it as a key therapeutic target.^20^ Fibrinogen‐like protein 2 (FGL2), a member of the fibrinogen‐like protein family, exerts regulatory influence over innate and adaptive immune responses.^21^ Yan et al. have demonstrated that FGL2 inhibits the differentiation of CD103+ dendritic cells (DCs) induced by granulocyte‐macrophage colony‐stimulating factor (GM‐CSF) by suppressing NF‐κB, STAT1/5 and p38 activation, consequently promoting the progression of brain tumours.^22^ Furthermore, Zhu et al. have uncovered that FGL2 induces the activation of cancer‐associated fibroblasts (CAFs), contributing to lung cancer progression.^23^ However, no studies have reported the expression levels and prognostic significance of FGL2 in BLCA. In current research, LST1, a gene encoding the MHC III region, has not been directly linked to tumour cells.^24^ Ceriani et al. reported that LST1 interacts with RalGPS2, facilitating the formation of tunnelling nanotubes (TNTs) in 5637 cells.^25^ In the context of the tumour microenvironment, the exchange of materials through TNTs confers specific characteristics upon cancer cells, including enhanced metabolic plasticity, migratory phenotypes, angiogenic capabilities and resistance to treatment.^26^

COL5A2, also known as the type V collagen alpha two chains, has previously been associated with poorer prognosis^27^ and increased invasiveness^28^ in BLCA. Liao et al. found that high IFITM2 levels in gastric cancer are linked to worse outcomes and reduced cell growth and spread when IFITM2 is silenced.^29^ Ma et al. showed that in elderly glioblastoma patients, higher IFITM2 expression and an inflammatory environment lead to poorer survival, with IFITM2 driven by IFN‐γ contributing to the aggressive cancer phenotype.^30^ Using Mendelian randomization, the study identified C1QB as a risk factor for colorectal cancer (CRC) and a promising drug target.^31^ Additionally, C1QB was found to be upregulated in cervical cancer tissues compared to benign and precancerous samples, correlating with disease severity and markers P16 and Ki‐67.^32^ C1QB may be involved in apoptosis, autophagy and drug sensitivity, suggesting it could be an oncogene in cervical cancer. Based on previous research, high IGFBP7 in BLCA is associated with aggressive features and poor immunotherapy response. It affects the tumour microenvironment and T cell activity, with an IGFBP7‐based model effectively predicting prognosis and treatment response.^33^ Annexin A5 (ANXA5) serves as a protein kinase C inhibitor and has been shown to play a role in the progression of various tumours beyond BLCA. In breast cancer, ANXA5 aids in membrane repair, essential for metastatic survival.^34^ In colorectal cancer, ANXA5 overexpression reduces cell migration and downregulates EMT‐related genes, hindering metastasis.^35^ In hepatocellular carcinoma, ANXA5 promotes cell migration, invasion and angiogenesis, correlating with poor prognosis.^36^ In the context of BLCA, elevated ANXA5 expression has been closely associated with worse disease‐free and progression‐free survival, suggesting its possible involvement in BLCA recurrence and progression.^37^ Our study confirmed the heightened ANXA5 expression in bladder tumours compared to normal tissues, and we observed that disrupting ANXA5 expression inhibited BLCA cell migration. These findings suggest that ANXA5 may play an important role in BLCA metastasis, and further investigation into the underlying mechanisms is warranted to better understand its function in this context.

Later, all patients were divided into low and high‐scoring groups using the scoring system we constructed. We found that the developed scoring system independently predicted overall survival in BLCA patients, with a worse prognosis observed in the high‐scoring group compared to the low‐scoring group. Clinical correlation analysis also indicated that patients in the high‐scoring group had higher Grades, T and N stages, consistent with our predictions. Additionally, we explored the relationship between Subtype A and B with the scoring system, revealing that Subtype A corresponded to higher scores. This could explain the poorer prognosis observed in Subtype A.

Immunotherapy has revolutionized cancer treatment, especially with the approval of immune checkpoint inhibitors targeting PD‐L1/CTLA‐4 in BLCA.^38^ However, not all patients benefit from immune checkpoint inhibitor therapy due to individual differences. The tumour immune microenvironment plays a crucial role in immunotherapy outcomes.^39^ Our study investigated the impact of a scoring system composed of cell differentiation‐related genes on the bladder tumour immune microenvironment. Using single‐cell subpopulation localization analysis from internal and external datasets, we observed that most genes in the scoring system were highly expressed in M2 macrophages. Subsequent transcriptome data analysis revealed a positive correlation between the expression of these genes and M2 macrophage expression. The consistency between our results from single‐cell and transcriptome data highlights the reliability of our analysis. M2 macrophages, also known as immunosuppressive cells and tumour‐associated macrophages, constitute a subset of cells in the tumour microenvironment.^40^ They can create an immunosuppressive environment characterized by the secretion of anti‐inflammatory cytokines such as IL‐10 and TGF‐β,^41^ inhibiting the activation of cytotoxic immune cells, including CD8+ T cells.^42^ This immunosuppression may hinder the natural anti‐tumour immune response of the body.^43^ The TCIA analysis suggests that in patients with positive PD1, higher CDR scores are associated with a better immunotherapy response. Conversely, in patients with negative PD1, lower CDR scores are linked to a better response. When PD‐1 positive patients show improved sensitivity to immunotherapy, it may be because immune checkpoint inhibitors can counteract PD‐1‐mediated suppression. Although high M2 macrophage infiltration may contribute to immune suppression, PD‐1 inhibitors might still be effective in addressing these challenges. Conversely, PD‐1 negative patients often respond better when M2 macrophage infiltration is low, as their immune environment may be less influenced by M2 macrophages, which could enhance the effectiveness of immunotherapy. Therefore, we suggest that for PD‐1 negative patients with high CDR scores who do not respond well to immunotherapy, adding drugs that target M2 macrophages might be beneficial.

While subtypes associated with cellular differentiation and the scoring system provide a certain insight into tumour prognosis and the immune microenvironment, it is essential to acknowledge certain limitations. This study analyzes publicly available single‐cell and transcriptomic data supplemented by basic in vitro experiments. Therefore, more sophisticated in vitro studies are warranted. Furthermore, the observed effect of ANXA5 expression interference, which impacts tumour cell migration without affecting proliferation, raises intriguing questions deserving of further in‐depth exploration. Additionally, the prediction of immune therapy responses remains retrospective and indirect, highlighting the need for prospective trials involving a larger patient cohort to enhance the reliability of the scoring system.

## 5 CONCLUSION

In this study, we have delineated a group of genes associated with cell differentiation in BLCA through single‐cell and WGCNA analysis. Leveraging these genes, we have identified distinct molecular subtypes linked to cell differentiation and introduced the cell differentiation‐related scoring system for prognosticating patient survival and assessing the immune microenvironment. Furthermore, we have identified the pivotal gene ANXA5 gene via comprehensive analysis and substantiated its involvement in enhancing tumour cell migration through in vitro experiments. This finding offers encouraging prospects for unveiling novel potential targets and therapeutic strategies in the context of bladder cancer treatment.

## AUTHOR CONTRIBUTIONS

**Sheng Li:** Conceptualization (equal); resources (equal); software (equal); supervision (equal); validation (equal); visualization (equal); writing – original draft (equal); writing – review and editing (equal). **Fucun Zheng:** Validation (equal); visualization (equal). **Zhipeng Wang:** Formal analysis (equal); funding acquisition (equal). **Situ Xiong:** Investigation (equal); methodology (equal); project administration (equal). **Jin Zeng:** Supervision (equal); validation (equal). **Songhui Xu:** Funding acquisition (equal); investigation (equal); supervision (equal); validation (equal); visualization (equal). **Bin Fu:** Conceptualization (equal); data curation (equal). **Xiaoqiang Liu:** Supervision (equal); validation (equal); visualization (equal); writing – original draft (equal); writing – review and editing (equal).

## FUNDING INFORMATION

This study was supported by the Jiangxi Provincial ‘Double Thousand Plan’ Fund Project (Grant Number. jxsq2019201027). National Natural Science Foundation of China (NO. 82260511) Jiangxi Provincial Natural Science Foundation for Distinguished Young Scholar (NO. 20232ACB216014). Jiangxi Provincial Natural Science Foundation (NO. 20212BAB216037, NO. 20232BAB206090).

## CONFLICT OF INTEREST STATEMENT

No conflict of interest.

## CONSENT TO PARTICIPATE

Patient participation was contingent upon informed consent.

## CONSENT FOR PUBLICATION

All authors have granted their consent for its publication.

## Supporting information


Figure S1.

Figure S2.

Figure S3.

Table S1.

Table S2.

Table S3.

Table S4.

Table S5.

Table S6.

## Data Availability

All data are from open access databases. TCGA database, GEO database and TCIA database.
