# Supplementary figures and images for: Integrated analysis of bulk and single‐cell RNA‐seq data reveals cell differentiation‐related subtypes and a scoring system in bladder cancer
Source: J Cell Mol Med. 2024 Oct 14;28(19):e70111. doi: 10.1111/jcmm.70111 (PMC11481023; doi:10.1111/jcmm.70111)

A

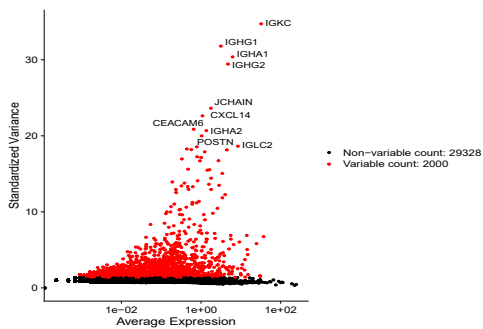

B

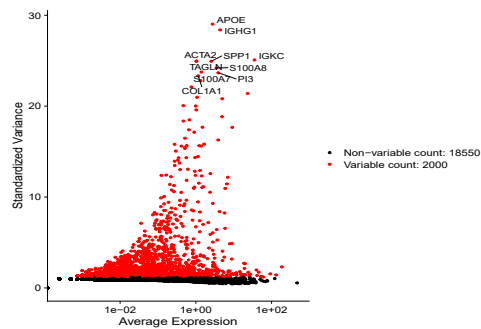

C

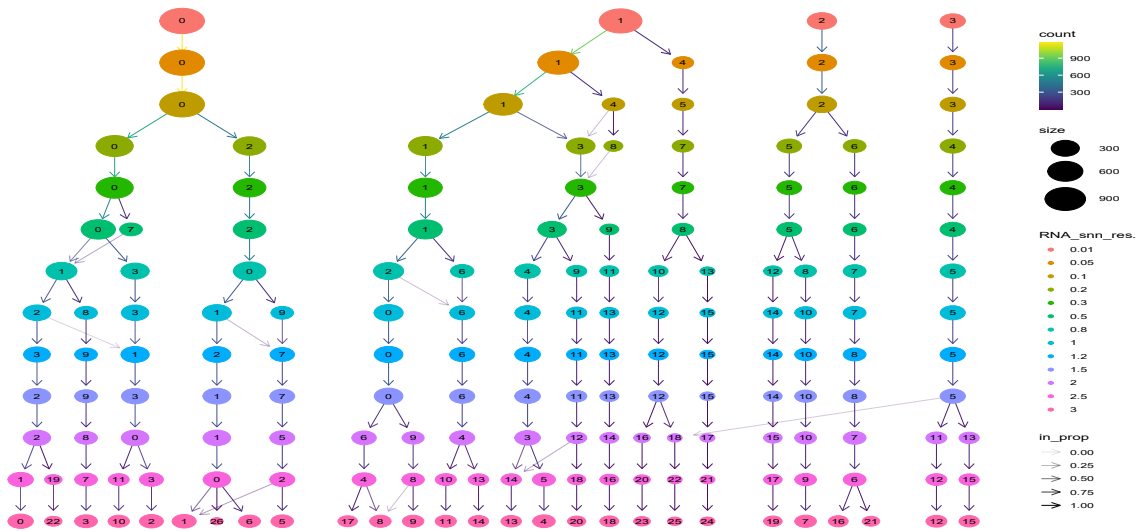

D

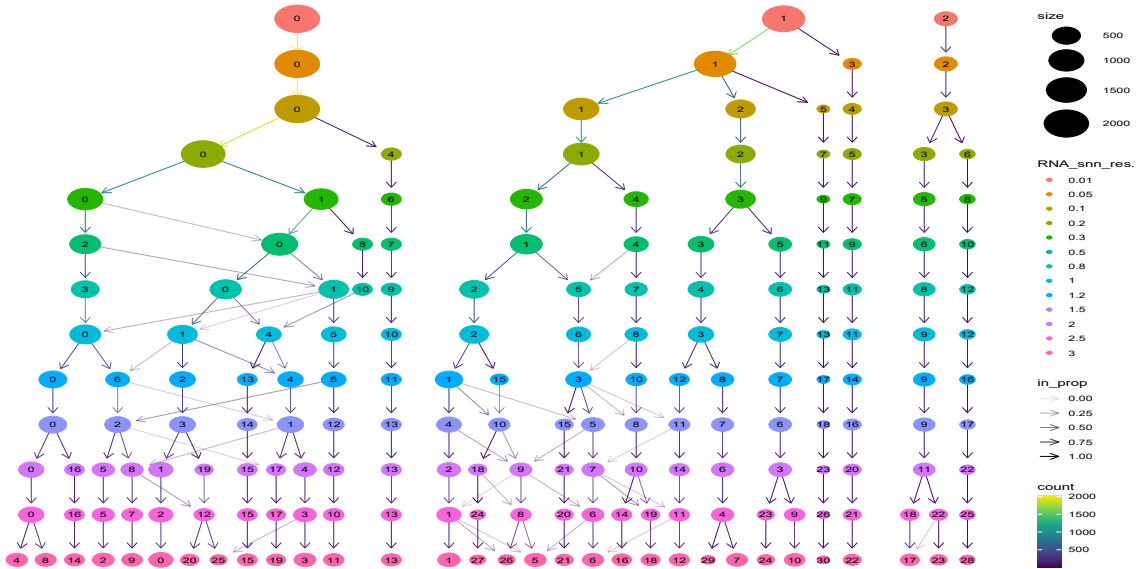

Supplement: Supplementary file 1 — Figure S1. [file JCMM-28-e70111-s001.pdf]

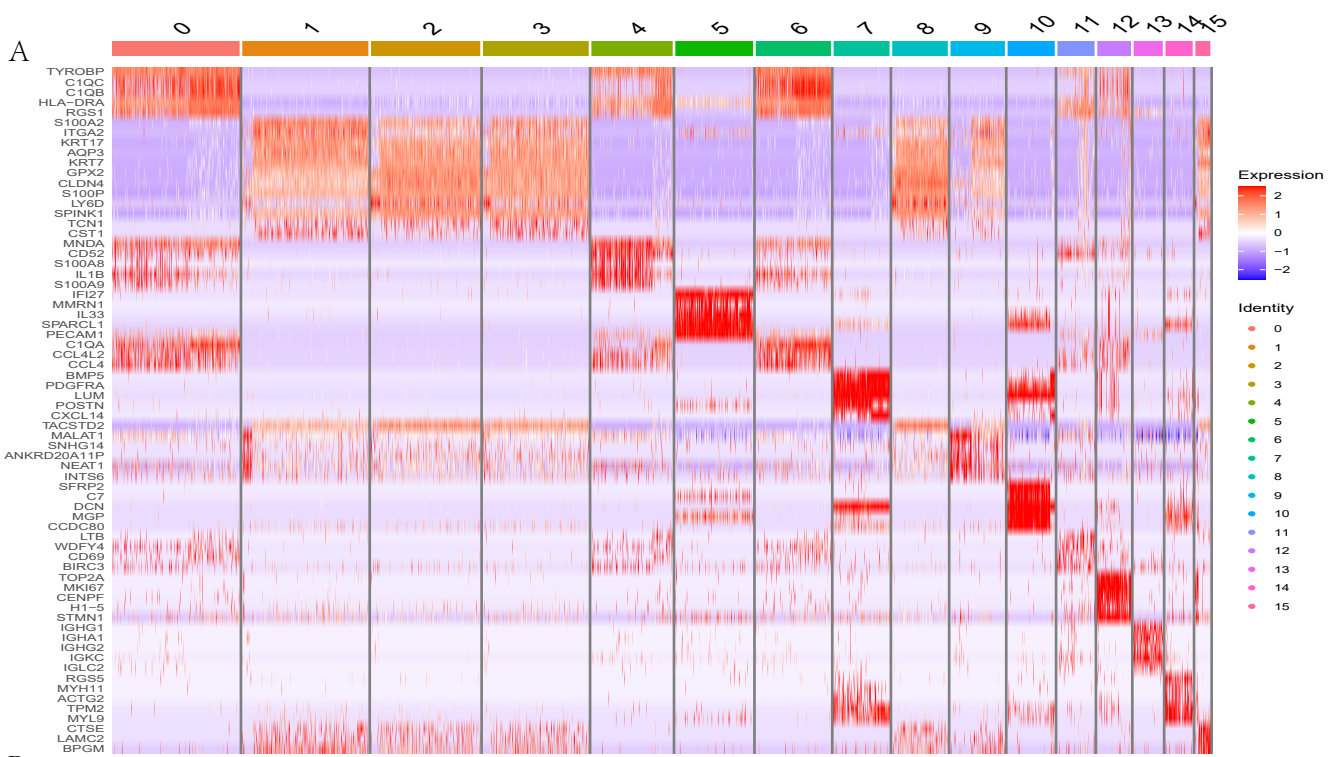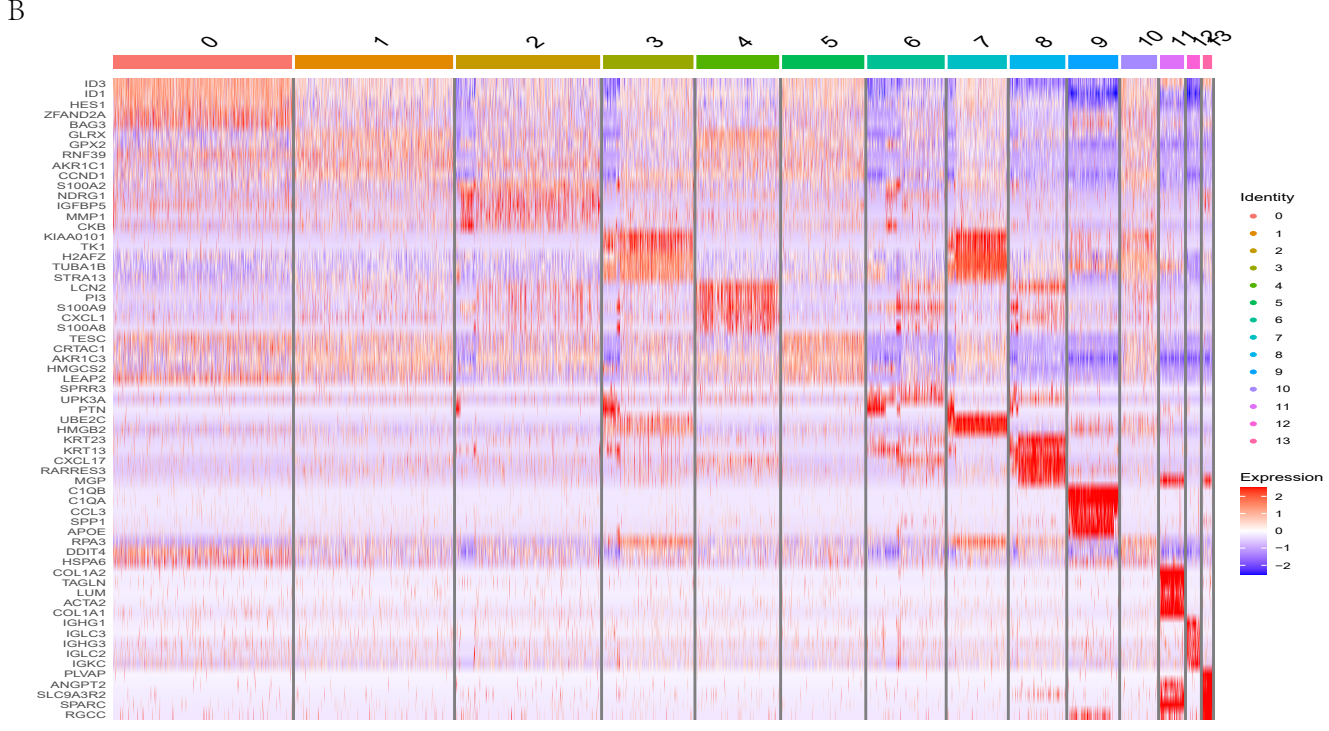

Supplement: Supplementary file 2 — Figure S2. [file JCMM-28-e70111-s009.pdf]

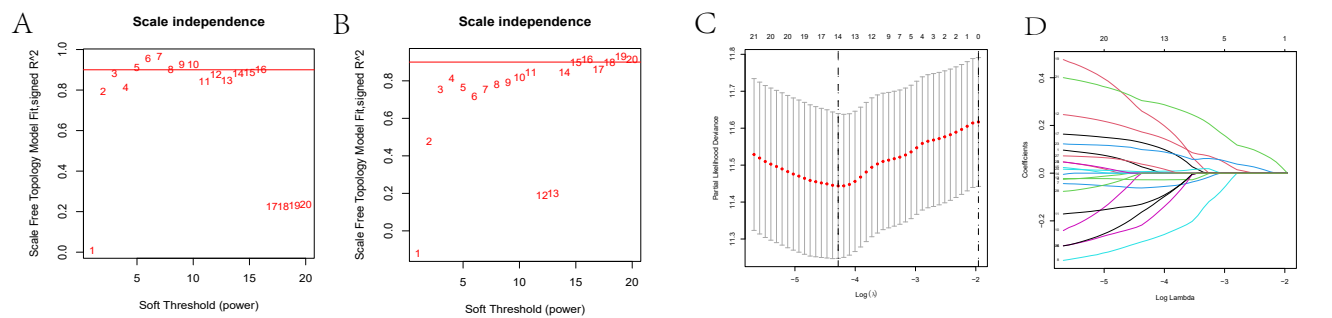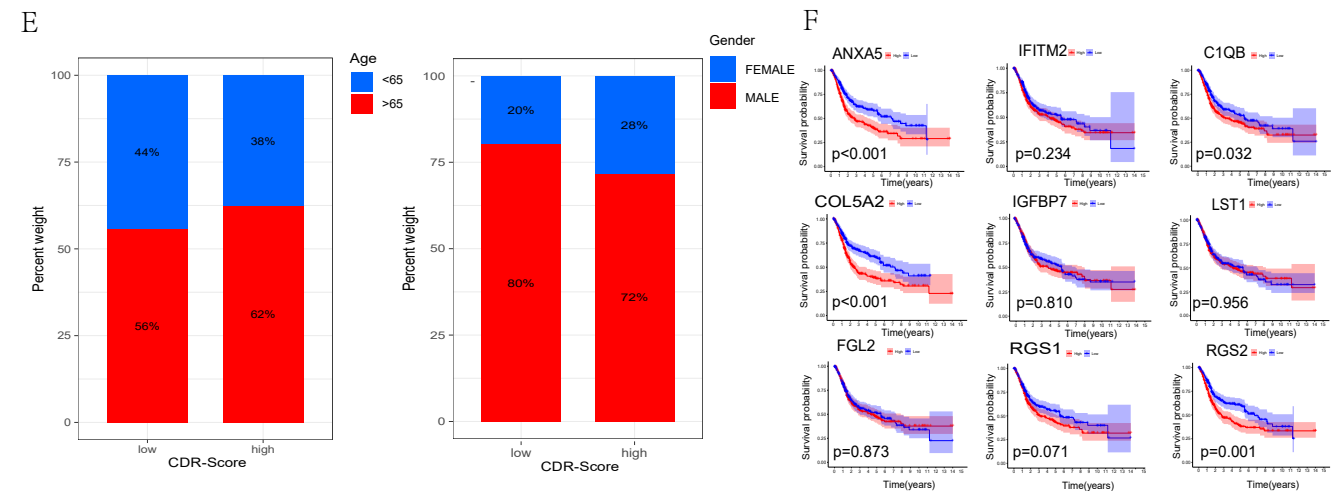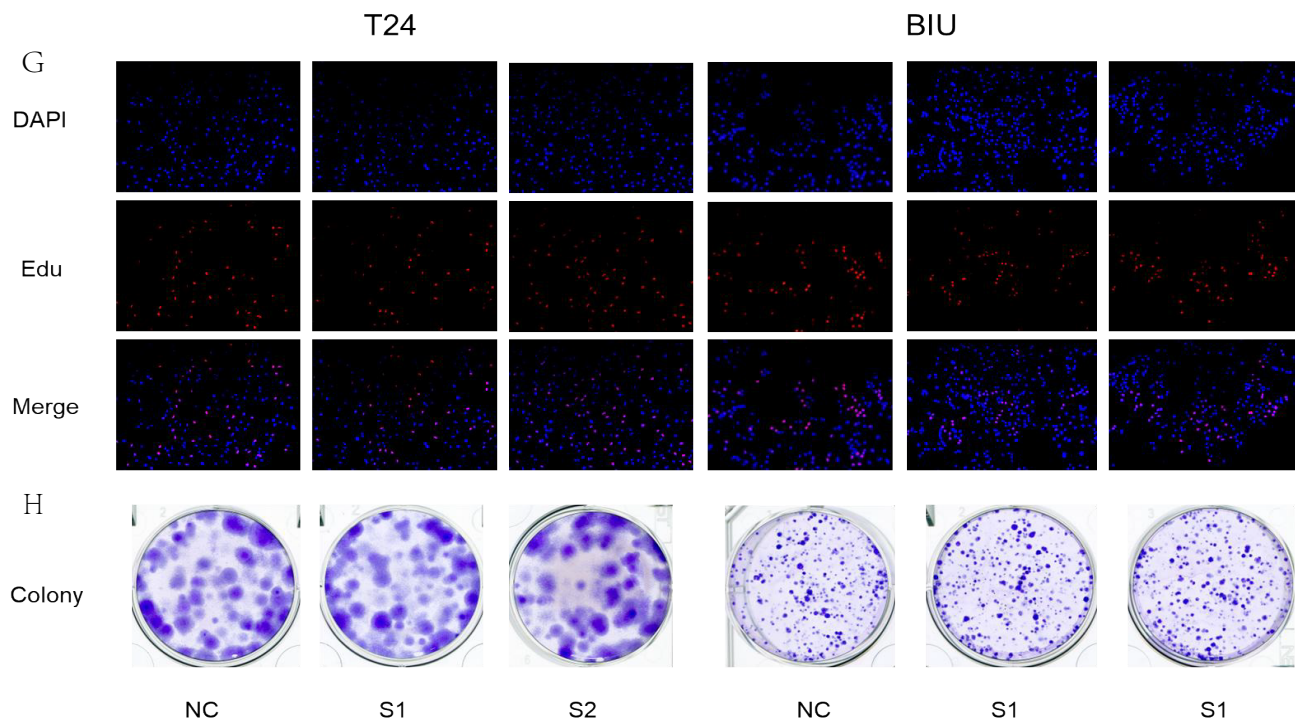

Supplement: Supplementary file 3 — Figure S3. [file JCMM-28-e70111-s007.pdf]
